# The immune regulatory role of exosomal miRNAs and their clinical application potential in heart failure

**DOI:** 10.3389/fimmu.2024.1476865

**Published:** 2024-12-02

**Authors:** Dandan Guo, Junchen Yan, Zhenyu Yang, Mengzhu Chen, Weibo Zhong, Xingxing Yuan, Siming Yu

**Affiliations:** ^1^ Department of Cardiology, Second Affiliated Hospital of Heilongjiang University of Chinese Medicine, Harbin, China; ^2^ School of Graduate Studies, Heilongjiang University of Chinese Medicine, Harbin, China; ^3^ Department of Gastroenterology, Heilongjiang Academy of Traditional Chinese Medicine, Harbin, China; ^4^ Department of Nephrology II, First Affiliated Hospital of Heilongjiang University of Chinese Medicine, Harbin, China

**Keywords:** heart failure, exosome, miRNA, immune modulation, macrophage polarization

## Abstract

Heart failure (HF) is a complex and debilitating condition characterized by the heart’s inability to pump blood effectively, leading to significant morbidity and mortality. The abnormality of immune response is a key factor in the progression of HF, contributing to adverse cardiac remodeling and dysfunction. Exosomal microRNAs (miRNAs) play a pivotal role in regulating gene expression and cellular function, which are integral to the crosstalk between cardiac and immune cells, influencing immune cell functions, such as macrophage polarization, T cell activity, and cytokine production, thereby modulating various pathological processes of HF, such as inflammation, fibrosis, and cardiac dysfunction. This review emphasizes the immune-regulatory role of exosomal miRNAs in HF and highlights their clinical potential as diagnostic biomarkers and therapeutic agents.

## 1 Introduction

Heart failure (HF) is a multifactorial syndrome characterized by the heart’s inability to pump sufficient blood to meet the body’s metabolic demands, remaining a leading cause of morbidity and mortality worldwide (1). The global prevalence of heart failure is estimated to be approximately 64 million people, with a prevalence rate of 1-2% in the general adult population (2). The morbidity and mortality rates of HF vary across different age groups, with a marked increase in the incidence and mortality as age advances. Among middle-aged adults, the incidence rate increases sharply, with estimates ranging from 4.7% to 13.3% (3). Compared with young adults, mortality rates are higher in middle-aged adults, older adults, and the elderly, with a 1-year mortality rate of 17.1%, 24.7%, and 39.9%, respectively (4). The pathophysiology of HF is complex and involves neurohormonal dysregulation, myocardial remodeling, and immunological abnormalities (5). The hyperactivity of renin-angiotensin-aldosterone system causes increased fluid retention and worsening cardiac function (6). Structural changes in the myocardium, such as fibrosis and hypertrophy, lead to ventricular stiffening and impaired relaxation (7). Among these factors, dysfunction of immune system plays a crucial role in the progression of HF, contributing to adverse cardiac remodeling, fibrosis, and ventricular dysfunction (8). With the progression of HF to advanced stage, patients fail to respond adequately to standard pharmacological treatments, often requiring additional interventions such as diuretics and inotropes, which can have limited long-term efficacy and potential adverse effects (9). Understanding the regulatory mechanisms governing immune modulation in HF is vital for developing novel therapeutic strategies.

Exosomes are small extracellular vesicles (EVs) ranging from 30 to 150 nm in diameter that are responsible for cell-to-cell communication (10). Exosomes are released by almost all cell types and carry diverse cargoes, including proteins, lipids, and nucleic acids, which reflect the physiological state of their cell of origin (11). Exosomal microRNAs (miRNAs), short non-coding RNA molecules that post-transcriptionally regulate gene expression by binding to target messenger RNAs (mRNAs), have emerged as critical regulators of gene expression and cellular function (12). During HF progression, exosomal miRNAs play crucial roles in various biological processes, including inflammation, apoptosis, and cellular remodeling, participating in cardiac hypertrophy and fibrosis (11). Moreover, their stability in circulation enhances the utility as reliable indicators of various pathological states of HF (13). Thus, exosomal miRNAs may serve as promising markers and therapeutic targets in HF. Notably, exosomal miRNAs are pivotal in orchestrating the complex interplay between cardiac and immune cells by modulating various immune cell functions, including macrophage polarization, dendritic cell (DC) maturation, T cell response, and cytokine production, thereby modulating the inflammatory response (14–16). Numerous studies have identified specific exosomal miRNA signatures associated with HF, suggesting their potential as non-invasive diagnostic biomarkers (17, 18). Furthermore, the therapeutic application of stem cell-derived exosomal miRNAs is an area of active investigation, as they have shown potential in preclinical models to reduce inflammation, mitigate fibrosis, and promote cardiac repair (19, 20).

This review concisely summarizes the biogenesis, processing, and functions of exosomes and exosomal miRNAs, as well as focuses on their immune regulatory roles of exosomal miRNAs in HF. It discusses the interplay between exosomal miRNAs and various immune cells, and their impact on the inflammatory milieu in HF. Additionally, it explores the clinical application potential of exosomal miRNAs, highlighting their potential as diagnostic biomarkers and therapeutic agents.

## 2 Biogenesis and biological functions of exosomes

The biogenesis of exosomes involves several molecular mechanisms that ensure proper formation, cargo loading, and release of EVs. This process begins with the formation of early endosomes, which mature into multivesicular bodies (MVBs). These MVBs contain intraluminal vesicles (ILVs) that are eventually released as exosomes upon fusion with the plasma membrane (21). The endosomal sorting complex required for transport (ESCRT) machinery, a multi-protein complex involved in the biogenesis of MVB, facilitates the sorting of ubiquitinated proteins into ILVs (22). Several specific molecules, such as tetraspanins and lipid rafts, contribute to the selective loading of cargo into exosomes, including specific proteins, lipids, nucleic acids, and other molecules, which ensure that exosomes carry distinct molecular signatures that dictate their functional roles (23). The transport and fusion of MVB are required for exosome release. The transport of MVBs to the plasma membrane is mediated by the cytoskeletal elements and motor proteins. Rab GTPases, particularly Rab35, are crucial regulators of MVB trafficking and fusion with the plasma membrane, facilitating exosome release (24). Moreover, fusion of MVBs with the plasma membrane is a tightly controlled process that involves soluble N-ethylmaleimide-sensitive factor attachment protein receptor (SNARE) proteins and other fusion machinery components, ensuring the timely release of exosomes into the extracellular space (25).

Exosomes exert pivotal biological functions in intercellular communication by transporting a diverse array of bioactive molecules between cells. Exosomes facilitate the transfer of metabolic and genetic information, thereby influencing the behavior and function of recipient cells. Their roles are particularly significant in immune modulation, inflammation, and cancer progression. For instance, exosomes can modulate immune responses by transporting antigens and other immune-related molecules, thereby influencing immune cell activation and response (26). They can also carry pro-inflammatory molecules that exacerbate inflammatory responses and are involved in the pathogenesis of inflammatory diseases (27). Additionally, exosomes are involved in cancer dynamics. They can promote tumor growth and metastasis by transferring oncogenic proteins and RNAs, facilitating processes such as epithelial-mesenchymal transition, and enhancing drug resistance (28). Thus, exosome-mediated communication by delivering its molecular cargo to recipient cells, plays a vital role in various physiological and pathological processes. While the cargo composition of exosomes is diverse and context-dependent, it is essential to consider the heterogeneity within exosome populations. Different subpopulations of exosomes can carry distinct sets of molecules that may exert various biological functions.

## 3 An overview of exosomal miRNAs

MiRNAs are small, non-coding RNA molecules that play critical roles in the regulation of gene expression. They have garnered significant attention owing to their involvement in various physiological and pathological processes, including cardiovascular diseases (29). Exosomes have emerged as crucial vehicles for intercellular communication and transport a variety of bioactive molecules, including miRNAs (30). Exosomal miRNAs have the potential to influence recipient cell function and behavior, making them important players in cell-to-cell communication and disease pathogenesis.

### 3.1 Biogenesis of miRNAs

**Figure 1:**
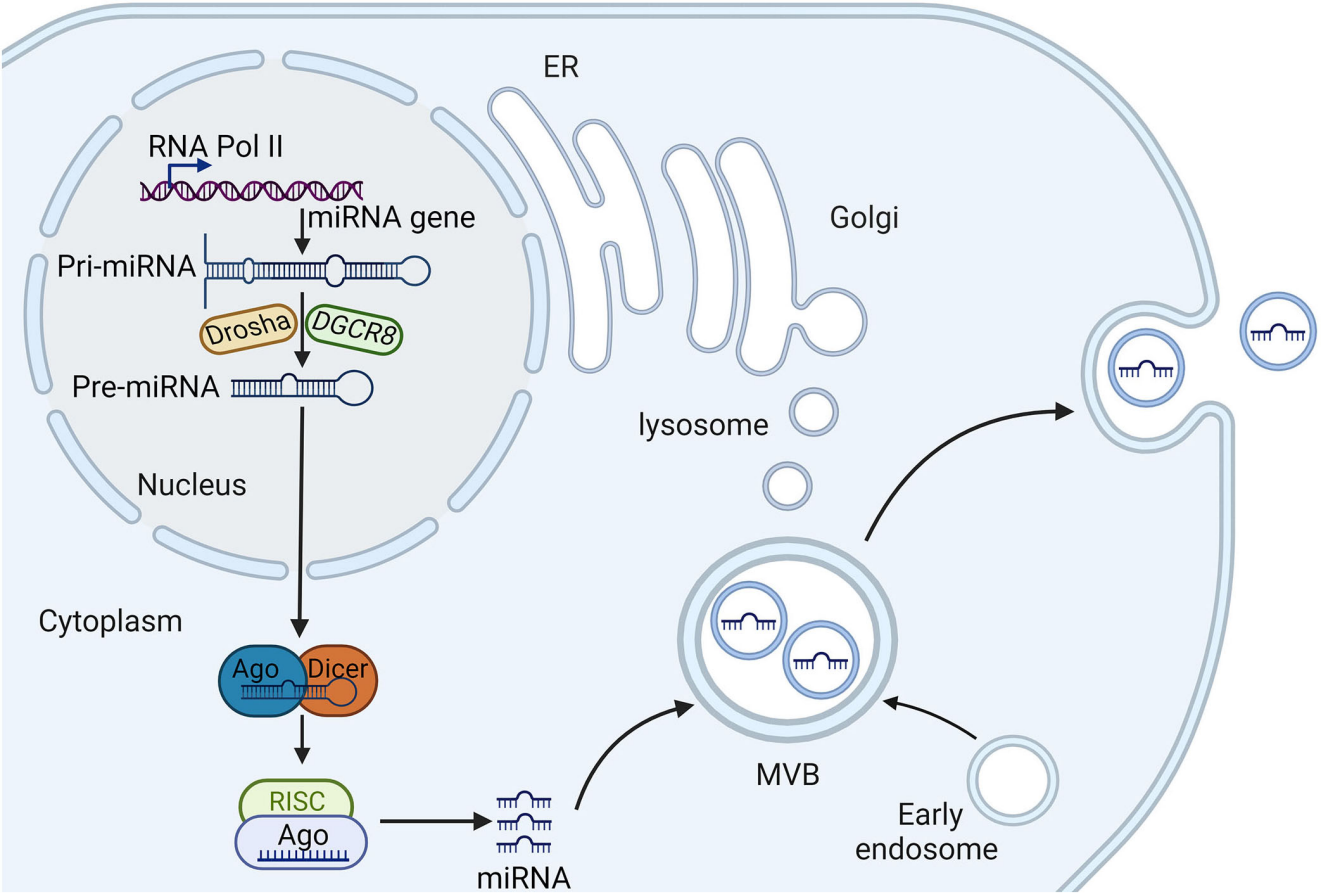
The biogenesis and sorting of exosomal miRNA. RNA polymerase II transcribes miRNA genes, generating pri-miRNA, which is processed in the nucleus by Drasha and DGCR8, producing pre-miRNA. Pre-miRNA is then transported to the cytoplasm and is further processed in the cytoplasm by Dicer, generating mature miRNA, which is incorporated into to silence the target gene. Early endosomes are generated by endocytosis of parent cell and then undergo the second invagination of the plasma membrane, thus forming, which are subsequently either degraded or released from the cell as exosomes. Several proteins are responsible for miRNA entry into the exosomes, such as heterogeneous nuclear ribonucleoproteins and neutral sphingomyelinase 2. miRNA, microRNA; AGO, argonaute; pre-miRNAs, precursor miRNAs; pri-miRNAs, primary miRNAs; RISCs, RNA-induced silencing complexes; MVBs, multivesicular bodies.

The biogenesis of miRNAs is a complex, multi-step process involving both canonical and non-canonical pathways, which are tightly regulated to ensure proper gene expression and cellular function (**Figure 1**). Initially, miRNAs are transcribed as primary miRNAs (pri-miRNAs) by RNA polymerase II, which are then processed in the nucleus by the microprocessor complex, consisting of the endoribonuclease Drosha and its cofactor DGCR8, resulting in precursor miRNAs (pre-miRNAs) (31). These pre-miRNAs are subsequently exported to the cytoplasm by Exportin-5, where they undergo further cleavage by another endoribonuclease, Dicer, to form miRNA duplexes (32). The miRNA duplexes are then loaded onto Argonaute (AGO) proteins to form the RNA-induced silencing complex (RISC), where one strand of the duplex is retained as mature miRNA, while the other strand is typically degraded (33). This mature miRNA within the RISC complex can then bind to complementary sequences in the 3′ untranslated regions (UTRs) of target mRNAs, leading to mRNA degradation or translational repression, thereby post-transcriptionally regulating gene expression (34).

In addition to the canonical pathway, non-canonical pathways also exist, in which miRNAs can be processed independently of Drosha, DGCR8, or Dicer. For instance, mirtrons are a class of miRNAs that bypass Drosha processing and are spliced directly from introns (35). The regulation of miRNA biogenesis is further modulated by various RNA-binding proteins (RBPs) and other regulatory factors that can enhance or inhibit different stages of miRNA processing. These factors include protein phosphorylation events that link miRNA biogenesis to cellular signaling pathways, and epigenetic modifications such as DNA methylation and histone modifications that influence the transcription and processing of miRNAs (36, 37). Additionally, the stability and turnover of miRNAs are controlled by mechanisms such as RNA methylation and uridylation, which can affect the half-life of miRNAs and their ability to regulate their target mRNAs (38). The biogenesis of miRNAs is not only crucial for normal cellular processes but also has significant implications in disease states. Understanding the detailed mechanisms of miRNA biogenesis, including both canonical and non-canonical pathways, and the regulatory networks involved, provides valuable insights into their roles in HF.

### 3.2 Exosomal miRNA sorting and transport

The sorting of miRNAs into exosomes is a highly selective and regulated process (**Figure 1**). Several main mechanisms have been proposed for the sorting of miRNAs into exosomes. The first is the involvement of specific RBPs such as heterogeneous nuclear ribonucleoproteins (hnRNPs) and Ago2, which recognize and bind to specific sequence motifs in miRNAs, facilitating their sorting into exosomes (39, 40). Furthermore, membranous proteins involved in EV biogenesis such as caveolin-1 and neutral sphingomyelinase 2, also play crucial roles in the selective sorting of miRNAs into EVs (41, 42). Besides, the selective loading of miRNAs into exosomes is mediated by specific sequence motifs. For example, the EXOmotif, a short sequence present in some miRNAs, has been identified as a determinant for miRNA sorting into exosomes (43). Components of the ESCRT complex, such as Alix and TSG101, have been implicated in the incorporation of specific miRNAs into exosomes (44).

Once exosomes are formed and loaded with miRNAs, they are released into the extracellular space through the fusion of MVB with the plasma membrane (45). Exosomal miRNAs are protected from degradation by RNases in the extracellular environment owing to their encapsulation within the lipid bilayer of exosomes. This protection enhances the stability of miRNAs, allowing them to be transported over long distances and to remain functional upon delivery to recipient cells (46). Exosomal miRNAs can then be taken up by recipient cells through various mechanisms, including endocytosis, phagocytosis, and direct fusion with the plasma membrane (47). The specific mechanism of uptake depends on the cell type and the surrounding microenvironment.

### 3.3 Exosomal miRNA functions

Exosomal miRNAs can regulate gene expression in recipient cells by binding to complementary sequences in target mRNAs, leading to mRNA degradation or translational repression (48). This regulatory function allows exosomal miRNAs to influence various cellular processes, including proliferation, differentiation, apoptosis, and immune responses (49). Besides, exosomal miRNAs play a ligand-like role in recipient cells, in which they function as direct agonists of specific receptor families by interacting with proteins, thereby affecting cellular processes and disease progression (50). Therefore, exosomal miRNAs play a critical role in intercellular communication by transferring genetic information between cells, thereby modulating the behavior of recipient cells, contributing to pathological processes of various diseases. Understanding the biogenesis, sorting, transport, and function of exosomal miRNAs is essential for elucidating their roles in HF.

While the general biological processes for exosomal miRNA biogenesis and function are understood, the identification of sorting signals that determine which miRNAs are packaged into exosomes is still incomplete. Also, the precise mechanisms by which exosomal miRNAs mediate intercellular communication and influence recipient cell behavior are not fully understood.

## 4 Immunomodulatory roles of exosomal miRNAs from different cell types

**Figure 2:**
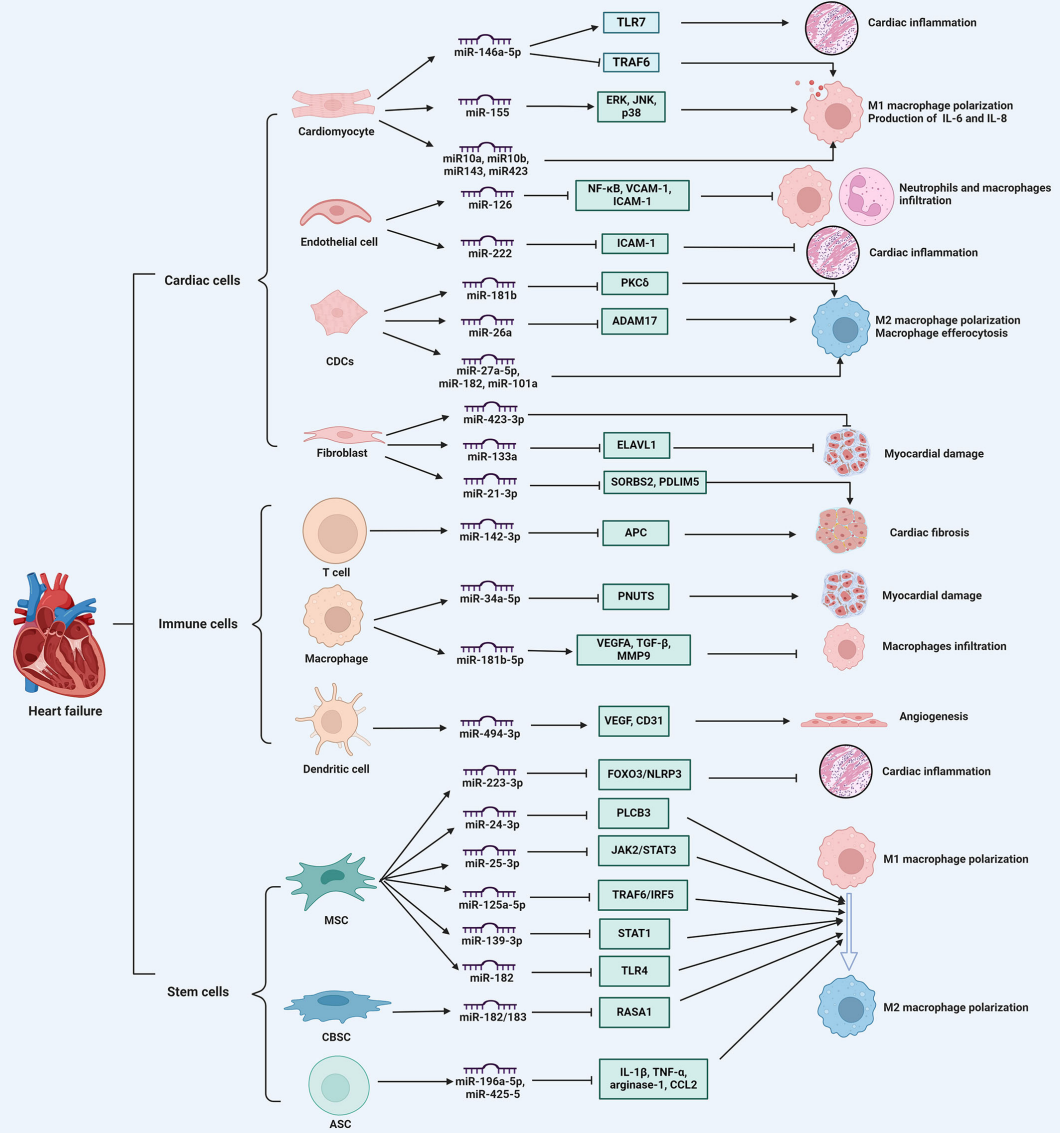
The immune regulatory role of exosomal miRNAs in heart failure. exosomal miRNAs derived from various cell types, including cardiac cells, immune cells, and stem cells, regulate immune cell infiltration, cytokine secretion, macrophage phenotype switch, thereby influencing myocardial inflammation, cardiomyocyte apoptosis, angiogenesis, and cardiac fibrosis, ultimately participating in the progression of heart failure. ADAM17, a disintegrin and metalloproteinase 17; Ang II, angiotensin II; APC, adenomatous polyposis coli; ASCs, adipose-derived stem cells; CBSCs, cortical bone stem cells; CDCs, cardiosphere-derived cells; ELAVL1, drosophila like RNA-binding protein 1; ERK, extracellular signal-regulated kinase; IL, interleukin; JNK, c-Jun N-terminal kinase; MSCs, mesenchymal stem cells; NFAM1, NFAT activating molecule 1; PDLIM5, PDZ and LIM domain 5; PKCδ, protein kinase C δ; PNUTS, serine/threonine-protein phosphatase 1 regulatory subunit 10; RASA1, ras p21 protein activator 1; SORBS2, sorbin and SH3 domain-containing protein 2; STAT1, signal transducer and activator of transcription 1; TLR4, toll-like receptor 4; TRAF6, TNF receptor-associated factor 6. ⊥ indicates an inhibitory effect and → indicates a promoting effect.

Exosomal miRNAs play significant immunomodulatory roles in HF by influencing various pathological processes, such as myocardial hypertrophy, injury, infarction, and ventricular remodeling. Various cell-derived exosomal miRNAs are involved in modulating immune responses, inflammation, and tissue repair by regulating the expression of genes related to various cellular processes and signaling pathways in HF (**Figure 2**).

### 4.1 Cardiac cells

Exosomal miRNAs from cardiac cells play a crucial role in modulating immune responses during HF progression. These miRNAs are involved in mediating intercellular communication and influencing immune cell behavior. For example, miR-146a-5p, when encapsulated in exosomes, is capable of activating various cardiac cell types, including cardiac fibroblasts and cardiomyocytes. This activation contributes to myocardial inflammation and cardiomyocyte dysfunction through the engagement of Toll-like receptor (TLR) 7 in nucleic acid sensing. Consequently, this process leads to the activation of immune cells, such as CD45^+^ leukocytes, monocytes, and neutrophils, as well as the subsequent production of proinflammatory cytokines, including C-X-C motif ligand (CXCL) 2, interleukin (IL)-6, and tumor necrosis factor (TNF)-α, which exacerbates cardiac inflammation (14). Indeed, compared to healthy individuals, the level of exosomal miR-146a-5p in patients with myocardial infarction is decreased. Further mechanistic evaluations revealed that exosomal miR-146a-5p derived from cardiomyocytes can modulate macrophage polarization, promoting M1 macrophage polarization and inhibiting M2 polarization, which in turn induces an inflammatory reaction; whereas, miR-146a-5p can downregulate the expression of TNF receptor-associated factor 6 to exert an anti-inflammatory effect (51). Similarly, when cardiomyocytes are exposed to hypoxic conditions, exosomal miRNAs, including miR-10a, miR-10b, miR-143, and miR-423, are elevated to induce IL-6 expression in macrophages, thus aggravating cardiac inflammation (52). Exosomes from hypertrophic cardiomyocytes also play a role in immune modulation by triggering the secretion of inflammatory cytokines IL-6 and IL-8 in macrophages. Notably, miR-155 has been identified as a crucial factor in initiating the inflammatory response, as it activates key signaling pathways such as extracellular signal-regulated kinase (ERK), c-Jun N-terminal kinase (JNK), and p38 (53). Consequently, these findings suggest that exosomal miRNAs influence macrophage phenotype and behavior to modulate immune responses, contributing to the repair process of infarcted myocardium.

Exosomal miRNAs from cardiosphere-derived cells (CDCs) are involved in modulating immune responses in HF. It has been shown that CDCs-derived exosomal miRNAs carry clusters of immune-related and cardiac-related molecular biomarkers, suggesting their significant roles in immune modulation and cardiac repair (54). In rat and pig models of myocardial infarction, exosomal transfer of miR-181b from CDCs into macrophages can modify their polarization state and confer cardioprotective efficacy by reducing the expression of protein kinase C δ (55). Following ischemic myocardial injury, exosomal miR-26a secreted by CDCs polarizes macrophages toward the phenotype with enhanced phagocytic capacity, which enhances macrophage efferocytosis and cardioprotection by sustaining the expression of MerTK and complement factor C1qa, a phagocytosis facilitator (56). Likewise, exosomes from CDCs attenuate macrophage infiltration and myocardial infarct size, and improve cardiac function by transferring miR-27a-5p, miR-182, and miR-101a to macrophages and regulating M2 macrophage polarization after myocardial ischemia/reperfusion (I/R) injury (57). Overall, exosomal miRNAs from CDCs play multifaceted roles in modulating immune responses, exhibiting their potential therapeutic applications in the management and treatment of HF.

Additionally, exosomal miRNAs derived from endothelial cells modulate immune cell functions and affect various pathological processes of HF. It is reported that miR-222 from endothelial microparticles reduces the expression of intercellular adhesion molecule (ICAM)-1 in recipient endothelial cells, which diminishes monocyte adhesion and promotes anti-inflammatory effects, protecting against cardiac dysfunction from myocardial infarction (58). Moreover, in septic mice, elevated miR-126 levels in endothelial cells mitigate severe cardiomyopathy and cardiac dysfunction by reducing the expression of adhesion molecules vascular cell adhesion molecule 1 and ICAM-1, thus further decreasing the accumulation of immune cells in the myocardium and inflammatory cytokine secretion (59). Hence, endothelial cell-derived exosomal miRNAs play an immunomodulatory role in influencing immune cell behaviors and cardiac inflammation during HF progression.

Fibroblast-derived exosomal miRNAs also regulate cardiac remodeling and fibrosis in HF. For instance, miR-21-3p, a passenger strand miRNA enriched in fibroblast-derived exosomes, induces cardiomyocyte hypertrophy by targeting proteins such as sorbin and SH3 domain-containing protein 2 and PDZ and LIM domain 5, suggesting its involvement in pathological cardiac remodeling (60). During the acute phase of ischemia-reperfusion injury, miR-423-3p expression is enhanced in fibroblast-derived exosomes, which increases cell viability and reduces apoptosis in cardiomyocytes under hypoxia/reperfusion condition (61). Further studies revealed that fibroblast-derived exosomal miR-133a protects cardiomyocytes against myocardial ischemia/reperfusion-induced injury through suppression of pyroptosis by suppressing the expression of drosophila like RNA-binding protein 1 (62). Hence, fibroblast-derived exosomal miRNAs predominantly exhibit cardioprotective effects, their dysregulation can contribute to pathological remodeling and cardiac fibrosis in HF.

### 4.2 Immune cells

Immune cells, particularly macrophages and DCs, have been implicated in the progression of HF through their involvement in inflammation and tissue remodeling (63, 64). In addition to resident immune cells, peripheral immune cells are recruited to participate in inflammatory processes and pathological remodeling in response to cardiac injury (65). In chronic HF patients, overexpressed exosomal miR-126 in plasma is associated with increased levels of IL-10, ICAM-1 and TNF-α in activated peripheral blood mononuclear cells (PBMCs) (66). In fact, circulating exosomes from experimental autoimmune myocarditis selectively load abundant miR-142, which causes CD4^+^ T cell immunometabolic dysfunction and exacerbates cardiac injury via repressing the methyl-CpG binding domain protein 2 and suppressor of cytokine signaling 1 (15). Also, in an acute myocardial infarction mouse model, exosomal miR-342-3p is suppressed to increase the expression of NFAT activating molecule 1, which is linked to DC maturation and cardiac inflammation (16). Besides, circulating exosomal miRNAs derived immune cells are overexpressed in HF patients, indicating the activation of peripheral immune system. These exosomal miRNAs, particularly those secreted by monocytes, macrophages, DCs, and T cells, are correlated with disease severity during HF progression (67). Thus, circulating exosomal miRNAs derived from immune cells can regulate immune responses and influence disease outcomes.

Macrophages, derived from tissue-resident cells or circulating monocytes, undergo significant phenotypic changes following cardiac injury, playing crucial roles in the inflammatory response and tissue repair in HF (68). The polarization of macrophages into pro-inflammatory (M1) and anti-inflammatory (M2) types is regulated by exosomal miRNAs, which can either exacerbate or mitigate inflammation and subsequent cardiac damage (69). For instance, during I/R-induced myocardial injury, exosomal miR-155-5p activates the intracellular JAK/STAT1 pathway in macrophages, promoting M1 macrophage proliferation and exacerbating cardiac inflammation (70). Also, in I/R-induced pig models of myocardial infarction, exosomal miR-181b-5p from M2 macrophages suppresses the number of CCR2^+^ macrophages in favor of the polarization of macrophages toward the M2 phenotype, which facilitates the migration and pro‐angiogenic vascular endothelial growth factor (VEGF) expression by endothelial cells, leading to improved angiogenesis and cardioprotective effect (71). Similarly, the upregulated levels of exosomal miR-34a-5p in programmed cell death 1 inhibitor-treated macrophages can mediate cardiomyocyte injury and cardiac senescence through inhibiting the expression of serine/threonine-protein phosphatase 1 regulatory subunit 10 (72). These findings imply that exosomal miRNAs derived from macrophages mediate inflammatory responses and myocardial damage, which are key factors in adverse cardiac remodeling and HF progression. In addition, as key modulators of immune responses and inflammation, DCs are antigen-presenting cells that contribute to systemic immune surveillance and regulate the inflammatory response during the development of cardiac fibrosis in HF (73). It has been demonstrated that exosomal miR-494 3p derived from DCs enhances the expression of VEGF in the infarcted myocardium of myocardial infarction (MI) model mice, which facilitates tube formation by cardiac microvascular endothelial cells, thereby promoting angiogenesis post-MI (74). Moreover, studies have shown that CD4^+^ T cells in HF patients exhibit increased activation and clonal expansion, which is associated with elevated levels of pro-inflammatory cytokines such as IL-6 and TNF-α, further contributing to adverse cardiac remodeling and sustained inflammation (75). Exosomal miRNAs from CD4^+^ T cells play a crucial role in the development of HF. For example, exosomes derived from activated CD4^+^ T cells have been shown to evoke pro-fibrotic effects in cardiac fibroblasts, thereby aggravating cardiac fibrosis and dysfunction post-infarction. This is primarily mediated through miR-142-3p, which is enriched in exosomes from CD4^+^ T cells and targets the adenomatous polyposis coli gene, a negative regulator of the WNT signaling pathway, thus promoting cardiac fibroblast activation and fibrosis (76). Overall, the evidence strongly supports the crucial role of exosomal miRNAs from immune cells, including macrophages, DCs, and CD4^+^ T cells in the development and progression of HF, making them a focal point for future research and clinical applications in this disease.

### 4.3 Stem cells

Stem cells, particularly mesenchymal stem cells (MSCs), have emerged as a promising therapeutic option for improving cardiac function in patients with HF. They have been shown to promote angiogenesis and the formation of new blood vessels, which improves blood supply to the ischemic heart tissue and supports myocardial repair (19). Of importance, stem cells are involved in the paracrine effect, where they secrete various growth factors, cytokines, and exosomes that modulate the local cardiac environment, reduce inflammation, and inhibit apoptosis of existing cardiomyocytes (77). Exosomes secreted from MSCs can induce the differentiation of pro-inflammatory macrophages towards a pro-resolving phenotype, reduce generation of pro-inflammatory cytokines, and facilitate the resolution of inflammation within the myocardial infarct area (78). By entrapping MSC-derived exosomes onto the surface of the elastomeric scaffolds, the number of M2 macrophages is enhanced to improve myocardial regeneration during the inflammatory and wound healing responses (79). Thus, exosomes derived from stem cells play a significant role in modulating immune responses, which is crucial for the therapeutic management of HF.

Specifically, exosomal miRNAs from MSCs have shown promising cardioprotective effects by modulating macrophage polarization. For instance, exosomes derived from MSCs are confirmed to enhance M2 macrophage polarization and further reduce inflammation and infarct size in a mouse model of myocardial I/R, primarily through the action of miR-182, which targets the toll-like receptor 4 pathway (80). Similarly, exosomes from nicorandil-pretreated MSCs have been shown to promote cardiac repair post-myocardial infarction by upregulating miR-125a-5p, which inhibits the TRAF6/IRF5 signaling pathway, thereby enhancing M2 polarization (81). Additionally, exosomes from human umbilical cord MSCs are verified to improve cardiac function by increasing M2 macrophage polarization through the downregulation of phospholipase C beta 3 and inactivation of the nuclear factor kappa B (NF-κB) pathway, mediated by miR-24-3p (82). These findings suggest exosomal miRNAs from MSCs can promote M2 polarization and reduce cardiac inflammation, further aiding heart repair in HF. Furthermore, exosomes from MSCs repress M1 polarization by miRNA-mediated cellular signaling and stress responses in HF. It is reported that bone marrow MSC-derived exosomal miR-25-3p confers cardioprotective effects in I/R-induced MI mice and downregulates proinflammatory cytokines in LPS-stimulated macrophages by inactivating the JAK2/STAT3 signaling pathway and subsequent inhibiting M1-like macrophage polarization (83). Consistent with this result, exosomal miR-139-3p from MSCs reduces the amount of M1 macrophages and facilitates cardiac repair in acute MI through suppressing downstream signal transducer and activator of transcription 1 (84). Of interest, in addition to macrophages, MSC-derived exosomal miRNAs like miR-223-3p can also be transferred into cardiomyocytes to inhibit LPS-induced cardiomyocyte inflammation and pyroptosis, which helps alleviate immune cell infiltration, inflammatory cytokine secretion, and cardiac dysfunction (85). Therefore, exosomal miRNAs derived from MSCs exert immunomodulatory effects via affecting the macrophage polarization and ameliorating cardiac inflammation in HF.

Moreover, other stem cells, like cortical bone and adipose stem cells, is verified to secrete exosomal miRNAs to exert cardioprotective effects in HF. In the myocardium post-MI model mice, cortical bone stem cell-derived exosomes which are enriched in miR-182/183, promote the polarization of macrophages and T cells towards a pro-reparative phenotype, which enhances oxidative phosphorylation rate and reduces reactive oxygen species (ROS) via the Ras p21 protein activator 1 axis, thereby improving structural remodeling and cardiac function (86). Besides, exosome-derived miR-196a-5p and miR-425-5p from adipose stem cells halt ischemia-induced mitochondrial dysfunction and ROS production in cardiomyocytes, and promote angiogenesis by polarizing macrophages toward the anti-inflammatory M2 immunophenotype (87).

In conclusion, exosomal miRNAs derived from various cell types, including cardiac cells, immune cells, and stem cells, represent a versatile and potent class of molecules with potential to modulate immune responses and influencing HF outcomes (**Figure 2**). These exosomal miRNAs are involved in macrophage polarization, DC maturation, and T cell response, thereby modulating inflammatory reactions and cardiac remodeling by acting on various molecular targets and signaling pathways, highlighting their potential as multifaceted therapeutic agents. Identification of exosomal miRNA signatures as biomarkers for HF diagnosis and prognosis is a promising approach. Integrating exosomal miRNA profiling into clinical practice could enhance the precision of HF management. Exosomal miRNAs derived from various cell types, such as cardiomyocytes, fibroblasts, endothelial cells, and immune cells, exert immunomodulatory effects and further affect cardiac structures and functions in HF. However, the functional diversity of exosomal miRNAs in regulation of immune responses in HF requires further investigation. Besides, temporal changes in exosomal miRNA profiles during the progression of HF and their correlation with disease stages and outcomes are not thoroughly explored.

## 5 Clinical application potential of exosomal miRNAs in HF

### 5.1 Diagnostic markers

Exosomal miRNAs are stable within the circulatory system due to their encapsulation in EVs, which protect them from degradation, making them reliable candidates for diagnostic purposes (17). The analysis of exosomal miRNAs in plasma or serum holds significant promise as a non-invasive biomarker for the early diagnosis of HF (18). A comprehensive characterization of miRNAs in plasma exosomes from dilated cardiomyopathy (DCM) patients with chronic HF identified 92 differentially expressed miRNAs correlated with several enriched pathways, suggesting their potential roles in the pathogenesis and clinical management of DCM (13). Research on patients with acute HF owing to DCM revealed exosomal-derived miR-92b-5p is upregulated, highlighting its potential as a non-invasive diagnostic biomarker (88). Similarly, it is reported that levels of miR129-5p isolated from plasma microvesicles are inversely related to the degree of clinical HF in patients with univentricular heart disease, indicating that miR129-5p is a sensitive and specific biomarker for this disease independent of ventricular morphology or palliation stage (89). Besides, the diagnostic potential of exosomal miRNAs extends beyond HF to other cardiovascular complications, such as HF with preserved ejection fraction in diabetic patients, where miR-30d-5p and miR-126a-5p are found to be correlated with reduced cardiac output and exosomal expression, supporting their release from the heart and association with diabetic HF with preserved ejection fraction (90). Likewise, in HF patients with reduced ejection fraction, elevated circulating exosomal miR-92b-5p is positively correlated with left atrial diameter, left ventricular diastolic diameters, and systolic diameters, acting as a promising diagnostic biomarker for this disease (91). The identification of exosomal miRNAs like miR-7856-5p in HF patients with depressive symptoms further underscores its diagnostic value, showing high sensitivity and specificity for severe depressive symptoms among HF patients (92). Moreover, dysregulated exosomal miRNAs reflect pathological processes during HF progression. For example, it has been shown that plasma exosomal miR-425 and miR-744, which serve as negative regulators of cardiac fibrosis by suppressing TGF-β1 expression, possess the potential to be a biomarker to predict cardiac fibrosis and HF (93). Consistently, circulating exosomal miR-146a is upregulated in response to systemic inflammation, implying its potential role in reflecting the inflammatory state of HF (94). As for central inflammation in chronic HF, circulating exosomal let-7g-5p and let-7i-5p are confirmed to reduce the neuroinflammation, which is associated with sympathetic hyperactivity in chronic HF (95). Of interest, three p53-responsive exosomal miRNAs (miR-194, miR-34a, and miR-192), which function as circulating regulators of HF development via the p53 pathway, are elevated by the early convalescent stage of acute MI, underscoring their diagnostic value, with a developed three-circulating exosomal miRNA model predicting the risk of development of ischemic HF after acute MI (96).

Taken together, dysregulated exosomal miRNAs indicate their potential as diagnostic factors for HF (**Table 1**). However, there are still challenges in identifying optimal exosomal miRNA candidates for early diagnosis and screening in patients. A promising strategy is to apply next-generation sequencing to winnow the vital exosomal miRNAs in these patients with different disease conditions and stages. Thus, further clarification of exosomal miRNA-related markers could provide a new angle for prognostic indicators of HF.

**Table 1:** Exosomal miRNAs act as potential diagnostic markers in HF.

Exosomal miRNAs	Expression	Clinical relevance	Ref.
miR-92b-5p	Up	DCM-related acute HF	(88)
miR129-5p	Down	HF with univentricular heart disease	(89)
miR-30d-5p, miR-126a-5p	Down	Diabetic HF with preserved ejection fraction	(90)
miR-92b-5p	Up	HF with reduced ejection fraction	(91)
miR-7856-5p	Up	HF with depressive symptoms	(92)
miR-425, miR-744	Down	HF with cardiac fibrosis	(93)
miR-146a	Up	HF with systematic inflammation	(94)
let-7g-5p, let-7i-5p	Down	HF with neuroinflammation	(95)
miR-194, miR-34a, miR-192	Up	Ischemic HF with acute MI	(96)

### 5.2 Therapeutic targets

Exosomal miRNA-based therapy holds significant potential in reversing cardiac remodeling and improving cardiac function in HF patients. For instance, in an MI rat model, exosomes loaded with miR-126 and miR-146a mimics promote the expression of vascular endothelial growth factor and tube formation, which helps to decrease infarct size and facilitate angiogenesis, as well as reduces the production of collagen fibers and cardiac fibrosis (97). By delivering exosomal miRNA-21 into cardiomyocytes and endothelial cells, cell apoptosis and cardiac function impairment are inhibited in a preclinical MI animal model (98). Thus, exosomal miRNA-based therapy could revolutionize HF treatment by reversing cardiac remodeling and improving cardiac function.

Specifically, MSC-derived exosomes have shown promise in cardiac repair and regeneration owing to their ability to carry specific miRNAs that provide anti-apoptotic, anti-inflammatory, anti-fibrotic, and angiogenic effects within the infarcted heart (99). For example, miR-129-5p loaded into MSC-derived exosomes has been demonstrated to ameliorate oxidative stress, cell apoptosis, inflammation, and fibrosis in cardiomyocytes in mice with HF by inhibiting the TRAF3/NF-κB signaling pathway, thereby alleviating ventricular dysfunction and improving cardiac function (100). Following myocardial I/R injury, MSC-derived exosomes act as tools for the specific delivery of miR-181a, which exert immune-suppressing effect and mitigate cardiac inflammation (101). Moreover, exosomal miRNAs derived from MSCs also alleviate MI-mediated cardiac dysfunction, thus delaying HF progression. It is reported that miR-200b-3p overexpressing MSC-derived exosomes enhance cardioprotective abilities by reducing cardiomyocyte apoptosis, cardiac inflammation, and myocardial fibrosis by suppressing BCL2L11 in MI mice (102). Similarly, exosomes from MSCs containing miR-302d-3p have been shown to impede inflammation and cardiac remodeling and promote cardiac function recovery in MI-treated mice by diminishing hypoxic cardiomyocyte apoptosis via the BCL6/MD2/NF-κB axis (103). Also, human umbilical cord MSC-derived exosomes loaded with miR-223 can relieve myocardial fibrosis and inflammation infiltration, and induce angiogenesis through the P53/S100A9 axis, thereby promoting myocardial repair in MI rats (104). The therapeutic potential of MSC-derived exosomes extends beyond MI to sepsis-induced myocardial injury during HF progression. For instance, in CLP-induced septic mice, exosomal miR-223 derived from MSCs is transferred to cardiomyocytes, which represses inflammation and cell death via inhibiting the expression of SEMA3A and STAT3, alleviating sepsis-induced cardiac injury (105). In the same preclinical setting, bone marrow MSC-derived exosomal miR-141 is found to bind to ameliorate myocardial injury in septic mice through regulating the PTEN/β-catenin axis (106). Intriguingly, in severe acute pancreatitis-induced rats, miR-29a-3p can be transferred into cardiomyocytes by MSC-derived exosomes, which inhibits inflammatory responses and improves cardiac function to attenuate myocardial injury via inactivating the HMGB1/TLR4/AKT signaling pathway (107).

Collectively, exosomal miRNAs postpone the pathological processes of HF, including mitigating cell apoptosis, oxidative stress, and inflammatory response, thereby improving cardiac function (**Table 2**). Thus, exosomal miRNA-based therapies may be beneficial for HF treatment by transferring protective miRNAs into myocardial injury areas. Accumulating evidence supports the use of MSC-derived exosomal miRNAs as a novel and effective therapeutic approach to improve cardiac function in patients with HF, offering a new avenue for treatment that leverages the natural regenerative capabilities of stem cells and their exosomal cargo. Despite these promising results, challenges such as understanding the interaction mechanisms with host cells, distribution within the body, metabolism, and long-term safety need to be addressed to optimize the clinical application of exosomal miRNA-based therapies.

**Table 2:** Exosomal miRNAs derived from MSCs act as potential therapeutic targets in HF.

Exosomal miRNAs	Downstream targets	Outcomes	Ref.
miR-129-5p	TRAF3/NF-κB	Inhibit oxidative stress, cell apoptosis, inflammation, and fibrosis in cardiomyocytes	(100)
miR-181a	Unknown	Exert immune-suppressing effect and mitigate cardiac inflammation	(101)
miR-200b-3p	BCL2L11	Reduce cardiomyocyte apoptosis, cardiac inflammation, and myocardial fibrosis	(102)
miR-302d-3p	BCL6/MD2/NF-κB	Attenuate inflammation and cardiac remodeling and promote cardiac function recovery	(103)
miR-223	P53/S100A9	Relieve myocardial fibrosis and induce the angiogenesis	(104)
miR-223	SEMA3A, STAT3	Represses inflammation and cardiomyocyte death	(105)
miR-141	PTEN/β-catenin	Ameliorate myocardial injury	(106)
miR-29a-3p	HMGB1/TLR4/AKT	Inhibit inflammatory response and improve cardiac function	(107)

## 6 Conclusion and future directions

This review emphasizes the multifaceted roles of exosomal miRNAs derived from cardiac cells, immune cells, and stem cells in modulating immune responses, including macrophage polarization, DC maturation, and T cell response during HF progression. Owing to their stability in bodily fluids and their specific expression profiles in HF patients, exosomal miRNAs have shown promise as non-invasive biomarkers for early diagnosis of HF. The therapeutic potential of exosomal miRNAs lies in their ability to alleviate pathological processes of HF, such as apoptosis, inflammatory responses, fibrosis, cardiac remodeling and cardiac dysfunction at the molecular level. Stem cell-derived exosomes loaded with specific miRNAs could offer targeted therapies, reducing adverse immune reactions and promoting cardiac repair. Despite the potential, several challenges need addressing, including the efficient delivery of exosomal miRNAs, ensuring their stability and activity *in vivo*, and overcoming the potential for off-target effects. Future research should focus on a deeper understanding of the molecular mechanisms underlying the immunomodulatory functions of exosomal miRNAs. This includes identifying specific miRNA targets, understanding their interactions with immune cell receptors, and elucidating the signaling pathways involved. Besides, the development of standardized protocols for the isolation and characterization of exosomes and their miRNA cargo is crucial, which ensures reproducibility and comparability of results across different studies, facilitating the translation of research findings into clinical practice. Furthermore, extensive clinical studies are needed to validate the diagnostic potential of exosomal miRNAs. Large-scale, multi-center trials will help establish their clinical utility and reliability as biomarkers for HF. Importantly, the safety and efficacy of exosomal miRNA-based therapies must be thoroughly evaluated, including assessing potential off-target effects, immune reactions, and long-term outcomes.

## Glossary

AGO: argonaute
CXCL: C-X-C motif ligand
DC: dendritic cell
ERK: extracellular signal-regulated kinase
HF: heart failure
ICAM: intercellular adhesion molecule
I/R: ischemia/reperfusion
mRNAs: messenger RNAs
miRNAs: microRNAs
NF-κB: nuclear factor kappa B
pre-miRNAs: precursor miRNAs
RBPs: RNA-binding proteins
ROS: reactive oxygen species
TLR: toll-like receptor
VEGF: vascular endothelial growth factor
CDCs: cardiosphere-derived cells
DCM: dilated cardiomyopathy
ESCRT: endosomal sorting complex required for transport
EVs: extracellular vesicles
hnRNPs: heterogeneous nuclear ribonucleoproteins
IL: interleukin
JNK: c-Jun N-terminal kinase
MI: myocardial infarction
MSCs: mesenchymal stem cells
PBMCs: peripheral blood mononuclear cells
pri-miRNAs: primary miRNAs
RISC: RNA-induced silencing complex
TNF: tumor necrosis factor
UTRs: untranslated regions.
